# Surgical Site Preparation Using Alcohol with Chlorhexidine Compared with Povidone Iodine with Chlorhexidine Results in Similar Rate of Infection After Primary Total Joint Arthroplasty

**DOI:** 10.3390/antibiotics14020155

**Published:** 2025-02-05

**Authors:** Stefan J. Hanish, Mateo J. Kirwan, Nuanqiu Hou, Tori J. Coble, William M. Mihalko, Christopher T. Holland

**Affiliations:** 1 Department of Orthopaedic Surgery and Biomedical Engineering, The University of Tennessee Health Science Center-Campbell Clinic, 1400 S. Germantown Rd., Germantown, TN 38138, USA; shanish@campbellclinic.com (S.J.H.); mkirwan@campbellclinic.com (M.J.K.); cholland@campbellclinic.com (C.T.H.); 2 Campbell Clinic Foundation, 1211 Union Ave., Suite 520, Memphis, TN 38104, USA; nhou@campbell-foundation.org (N.H.); tori.coble@lmunet.edu (T.J.C.)

**Keywords:** surgical site preparation, periprosthetic joint infection, total joint arthroplasty

## Abstract

**Background:** Periprosthetic joint infection (PJI) is a devastating complication after total joint arthroplasty. A skin antiseptic solution is used to reduce the bacterial count and prevent PJI. There is no consensus in the literature on the application of antiseptic solutions. This study aims to compare the rate of infection between patients who received alcohol wash with Chloraprep to those who received povidone iodine wash with Chloraprep. **Methods:** A total of 607 patients who underwent total hip arthroplasty (THA) and total knee arthroplasty (TKA) at a single institution between January 2009 and July 2023 were reviewed. Perioperative variables were collected. The infection rate was used as a primary outcome. An odds ratio was calculated to compare infection and complication rates between the groups. **Results:** For patients who underwent THA, no difference in the rate of complications (alcohol wash: n = 6, 4.5%; povidone wash: n = 5, 3.6%; OR: 0.796; 95% CI: 0.237–2.673) or infection (alcohol wash: n = 1, 0.7%; povidone wash: n = 2, 1.4%; OR: 1.942; 95% CI: 0.174–21.667) was found. No difference in the rate of complications (alcohol wash: n = 3, 1.9%; povidone wash: n = 2, 1.2%; OR: 0.635; 95% CI: 0.105–3.849) or infection (alcohol wash: n = 0; povidone wash: n = 1, 0.6%; OR: 0.994; 95% CI: 0.983–1.006) was found in patients who underwent TKA. **Conclusions:** Surgical site preparation using alcohol wash with chlorhexidine offers similar short-term benefits in preventing postoperative infection to a povidone iodine wash with chlorhexidine in primary total joint arthroplasty. The use of alcohol wash and chlorhexidine is effective, while reducing the preparation time.

## 1. Introduction

Over 500,000 surgical site infections (SSIs) occur each year in the United States, accounting for 38% of nosocomial infections [1,2,3]. In total hip and knee arthroplasty (THA, TKA), the rate of surgical site infection has been reported to range from 0.3% to 1.9% for primary cases and up to 10% for revision surgery [3,4]. Despite the low rate of this complication, the morbidity that is associated with postoperative infection in total joint arthroplasty (TJA) is devastating. Postoperative infection imposes a significant physical, psychological, and financial burden, resulting in prolonged hospitalization, the need for readmission, revision surgery, delayed wound healing, prolonged antibiotic use, and increased costs [1,3,5,6]. Despite the severe complications that are associated with SSI, there is limited evidence to support standardized preoperative surgical site preparation in TJA.

The use of a skin antiseptic solution aims to reduce the bioburden of infective agents at the incision site to a level below that of the patients’ immune threshold [1]. Clinical practice guidelines reported by the Centers for Disease Control and Prevention (CDC) and World Health Organization (WHO) recommend surgical site preparation with alcohol-based solutions [7,8,9,10,11,12,13]. Chlorhexidine is most often cited as the superior antiseptic solution in the current literature, but much of this evidence stems from studies in general, urologic, and gynecologic surgery [3,14]. Across the subspecialties of orthopedic surgery, the recommendations differ or are largely nonexistent, as the evidence is limited and the anatomic location of the surgical site plays a significant role [3,11,15,16,17,18,19,20]. In the field of hip and knee arthroplasty, the topography of the hip and its proximity to distinct areas such as the inguinal fold, buttock, and perineum creates a unique bacteria profile for prep solutions to cover, with the most common bacteria being S. epidermidis, Corynebacterium, and M. Luteus [11].

The current literature regarding surgical site preparation in joint arthroplasty is limited. The existing literature should be interpreted with caution, as the formulation, concentration, and application of antiseptic solutions are inconsistent within and between studies [11]. This study compared the short-term infection rates of patients who received an alcohol wash with a chlorhexidine and alcohol product (Chloraprep, Becton Dickinson, Franklin Lakes, NJ, USA) versus a povidone iodine wash with Chloraprep for skin decontamination before primary TJA. We hypothesized that subjects who received an alcohol wash followed by Chloraprep would have reduced rates of postoperative SSI compared with those who received a povidone iodine wash followed by Chloraprep.

## 2. Results

A total of 607 patients who underwent primary TJA were studied. Of these patients, 281 (45.5%) underwent primary THA, and 336 (54.5%) underwent primary TKA. The mean age at the time of surgery was 69.1 years, and 336 (54.5%) were female. All patients received perioperative antibiotic prophylaxis consisting of cefazolin and vancomycin as per the hospital protocol for the standard of care based on the nosocomial bacterial profile for the institution. Clindamycin was given if a cefazolin allergy was reported. Antibiotic irrigants were used for intraoperative irrigation and transitioned to Irrisept (Irrimax, Gainesville, FL, USA) when the product became commercially available in 2010 for both groups. Wound vac and postoperative incision dressing were applied at the surgeon’s discretion. A StrataFix Knotless Tissue Control Device (Johnson and Johnson, New Brunswick, NJ, USA) was implemented in closure for both groups once it became commercially available in 2016.

Of the 607 patients, 298 (48.3%) patients received an alcohol wash with Chloraprep (THA: 135; TKA: 163), and 309 (50.1%) patients received a povidone iodine wash with Chloraprep (THA: 140; TKA: 169). The age was not significantly different between the two groups (alcohol: 69.1; betadine 69.2; *p* = 0.893). The numbers of patients who had comorbidities that increased their risk of PJI were not significantly different between the two groups (Table 1).

For patients who underwent THA, no difference in the rate of complications (alcohol wash: n = 6, 4.5%; povidone wash: n = 5, 3.6%; OR: 0.796; 95% confidence interval [CI]: 0.237–2.673) or infection (alcohol wash: n = 1, 0.7%; povidone wash: n = 2, 1.4%; OR: 1.942; 95% CI: 0.174–21.667) was found (Table 2). In the alcohol group, one patient experienced nondisplaced fracture of the lesser trochanter after a fall and was managed with conservative treatments; one developed superficial hematoma requiring surgical irrigation and debridement; one developed deep vein thrombosis that was treated with coumadin; one had subsidence of the femoral component and was managed with conservative treatments; one developed thrombosis requiring thrombectomy; and one developed a prosthetic joint infection, which was treated with antibiotic cement spacer placement and subsequent revision. The patient who developed PJI had a history of human immunodeficiency virus. In the povidone group, two patients experienced episodes of myocardial infarction postoperatively and were unable to be resuscitated; one developed superficial hematoma requiring surgical irrigation and debridement; one experienced a superficial soft tissue infection that was treated with irrigation and debridement; and one developed a PJI that was treated with irrigation and debridement and subsequent revision.

For patients who underwent TKA, no difference in the rate of complications (alcohol wash: n = 3, 1.9%; povidone wash: n = 2, 1.2%; OR: 0.635; 95% CI: 0.105–3.849) or infection (alcohol wash: n = 0; povidone wash: n = 1, 0.6%; OR: 0.994; 95% CI: 0.983–1.006) was found (Table 2). In the alcohol group, one patient developed persistent postoperative pain and repeated hemarthrosis requiring surgical irrigation and debridement; one developed significant skin necrosis; and one developed superficial hematoma requiring surgical irrigation. In the povidone group, one patient developed flexion contracture that required manipulation under anesthesia, and one patient developed a PJI that required a two-stage revision.

## 3. Discussion

This study showed that surgical site preparation with alcohol and Chloraprep provided similar short-term benefits to povidone iodine and Chloraprep in preventing postoperative infection after primary TJA. The recommendations for surgical site preparation in orthopedic surgery are inconsistent and vary between subspecialties. In the fields of foot and ankle, spine, and total joint arthroplasty, the evidence for a superior skin antiseptic agent is nonexistent [3]. Considering the devastating complications of PJI and the increasing frequency of TJA in the aging United States population, a standardized protocol for surgical site preparation is warranted [21].

This study demonstrated that alcohol and Chloraprep provide a similar reduction in SSIs to povidone iodine and Chloraprep in the setting of primary TJA. The core requirements of a preoperative skin antiseptic include rapid onset of action, broad-spectrum coverage, and persistent suppression of remaining organisms throughout the operation [22]. Povidone iodine is broad spectrum, has a persistent effect on bacterial growth, and effectively reduces microbial populations after application [22,23]. In orthopedic surgery, the literature on hand surgery, spine surgery, joint arthroscopy, and foot and ankle surgery supports the use of povidone iodine as an effective agent to prevent surgical site infection [3,19,24,25,26]. In this study, alcohol served as an alternative to povidone iodine. Alcohol has been shown to be an effective, broad-spectrum skin antiseptic that provides the most rapid reduction in bacterial count of all antiseptic solutions [3,22,27]. Despite its short duration of action, alcohol can be paired with longer-acting agents, such as chlorhexidine in our study, to provide sustained microbial suppression throughout the operation. A 2021 meta-analysis by Mastrocola et al. evaluated the efficacy of preoperative skin preparation with chlorhexidine–alcohol versus povidone iodine to reduce the normal bacteria flora in clean orthopedic surgery [20]. While they found both treatments to be effective at reducing bacterial loads, chlorhexidine–alcohol was shown to be superior to povidone iodine [20]. To the best of our knowledge, our study is the first to evaluate the specific preoperative skin antisepsis protocol of alcohol and Chloraprep compared with povidone iodine and Chloraprep. Our results showed no difference in the rate of infection or complications between cohorts at the 30- and 90-day postoperative follow-up. One case of PJI was identified in the alcohol wash group. The patient was at higher risk of infection due to a history of HIV. Therefore, this study demonstrated that the use of an alcohol wash with Chloraprep produced similar results or possibly a reduction in short-term surgical site infection compared with povidone iodine and Chloraprep during primary TJA.

Compared to other skin antiseptic agents, preparation with povidone iodine can be time-consuming and costly. As per manufacturer guidelines, povidone iodine requires a 3 to 5 min application time to be effective, with some traditional application techniques involving 5 to 7 min of preparation time [22,28]. Each additional minute in the operating room increases the cost of surgery and amount of time that the patient must spend under anesthesia. Furthermore, if this time requirement is not dutifully met, this risk of inadequate skin preparation can occur. In contrast to povidone iodine, alcohol dries quickly, avoiding inefficient and costly waiting periods [3]. As this study demonstrated similar outcomes with use of alcohol and povidone, using alcohol as an alternative, orthopedic surgeons can achieve a similar level of protection against surgical site infection while reducing the cost and operative time. As over half of primary TJAs are predicted to be performed at ambulatory surgery centers (ASCs) by 2026, efficiency and reducing operative time are important considerations [29]. Using alcohol as an alternative to povidone iodine is an effective strategy to meet certain state-sanctioned limits on the procedure length of ASC, such as Connecticut’s maximum operative time limit of 90 min [30]. Our study showed alcohol to be an effective, cost-saving alternative to povidone iodine in primary TJA.

A major drawback of alcohol as a preoperative skin antiseptic agent is its short duration of action [3,27]. Alcohol provides the most rapid reduction in bacterial load of all skin antiseptic agents, but dries quickly and loses its bactericidal activity once evaporated [3,27]. To remedy this shortcoming, alcohol can readily be combined with a longer-acting agent, such as chlorhexidine, to provide antimicrobial defense throughout the procedure [3,31]. Chlorhexidine possesses both bactericidal and bacteriostatic properties [3]. When compared with other skin antisepsis solutions, it has rapid action, persistent activity despite exposure to bodily fluids, and a residual effect after application [3]. Our use of alcohol and chlorhexidine followed clinical practice guidelines supporting alcohol-based solutions, as well as research on orthopedic surgery and general surgery supporting the use of chlorhexidine–alcohol versus povidone iodine [14,20].

Identifying the most effective skin antiseptic agent for TJA remains a critical priority in preventing surgical site infections. However, the modification of additional variables such as the timing and technique of the surgical site preparation process has been shown to have significant consequences in the rates of surgical site infections. Researchers have illustrated reduced rates of surgical site infection when investigating their institutional or personal surgical site preparation techniques. A 2016 case–control study showed that chlorhexidine skin preparation with gauze sponges produced significantly fewer unprepared areas of skin compared with commercially available prep-stick applicators in orthopedic hand procedures [32]. A 2016 prospective randomized, double-blind study showed significantly lower rates of SSI when a re-application of the skin prep solution after the final draping was performed in TJA [1]. Further research is needed to identify the most effective skin antiseptic agent and to explore surgeon- and institution-specific preparation techniques that could provide enhanced protection.

Our study was subject to several limitations. Its retrospective design carried the inherent limitations that are common to all retrospective investigations, including potential selection bias, incomplete data, and the inability to establish causality. Infections associated with TJA are rare, requiring large sample sizes to accurately characterize the pathology. While our sample size was large, the low frequency of this complication could have created limitations in its investigation. Our study’s primary outcome was the respective rates of infection following alcohol and povidone iodine skin preparation. At our institution, the primary author uses alcohol skin preparation, while other TJA surgeons utilize povidone iodine skin preparation. Therefore, an element of confounding bias was possibly introduced into the study, since one surgeon performed the intervention.

## 4. Materials and Methods

This retrospective cohort study aimed to investigate the incidence and associated risk factors for the development of postoperative infection after antiseptic skin preparation with either alcohol wash and Chloraprep or povidone iodine and Chloraprep. All procedures were performed by orthopedic surgeons at a single institution. Prior to study commencement, this work was approved by the Institutional Review Board (IRB) of University of Tennessee Health Science Center (23-09489-XP).

Primary total knee arthroplasty (TKA) and total hip arthroplasty (THA) cases that were performed at a single hospital between 2009 and 2023 were eligible for inclusion. During the study period, one surgeon utilized alcohol wash with chlorhexidine, while the other TJA surgeons utilized povidone wash with chlorhexidine. Patients who underwent alcohol and chlorhexidine skin prep were then identified from the single surgeon’s schedule and age-matched to a cohort of patients who underwent iodine and chlorhexidine skin prep. This resulted in 607 patients being included for analysis: 298 in the alcohol and chlorhexidine cohort and 309 in the iodine and chlorhexidine cohort. Subjects were excluded if the procedure was a revision total hip arthroplasty or revision total knee arthroplasty.

Data were collected from electronic medical records. Collected variables included the patients’ demographic information, surgical details, preoperative antibiotics, preoperative skin preparation, intraoperative irrigation, surgical site closure, postoperative dressing, and postoperative complications. Procedure-related variables that were recorded included preoperative antibiotic, preoperative skin antiseptic, intraoperative irrigation, method of incision closure, application of wound vacuum, and postoperative incision dressing. Follow-up data were collected at two intervals, 30 and 90 days post-operatively.

The primary outcome assessed was the frequency of postoperative surgical site infection, with stratification across total hip arthroplasty and total knee arthroplasty.

Descriptive statistics were used to summarize the demographic, preoperative, intraoperative, and postoperative data. Continuous variables were reported as means ± standard deviations or medians with interquartile ranges, depending on the distribution. Categorical variables were reported as frequencies and percentages. Odds ratios were calculated using SPSS (Version 29.0.2.0, Armonk, NY, USA: IBM Corp), with a significance level set at α = 0.05.

## 5. Conclusions

Our study demonstrated that surgical site preparation using an alcohol wash combined with Chloraprep offered comparable short-term benefits to povidone iodine and Chloraprep in preventing postoperative infections following TJA. Alcohol requires less time to dry when compared with povidone, which reduces the preparation time. Minutes saved from skin preparation may reduce surgical cost, decrease the amount of time that the patient is under anesthesia, and increase the efficiency of the ambulatory surgery center. Implementing a standardized preoperative skin preparation protocol with alcohol and chlorhexidine proved effective in minimizing the risk of surgical site infections while being cost-efficient by eliminating the need for extended preparation times.

## Figures and Tables

**Table 1:** The numbers of patients who had comorbidities that may increase the risk of PJI were not significantly different between the alcohol wash group and the povidone wash group.

THA			
Comorbidity	Alcohol Cohort, n (%)	Povidone Cohort, n (%)	*p*-Value
Rheumatoid Arthritis	12 (8.9)	17 (12.1)	0.44
Class III Obesity	7 (5.2)	17 (12.1)	0.05
Tobacco Use	36 (26.7)	30 (22.2)	0.33
Diabetes Mellitus	20 (14.8)	22 (15.7)	0.87
HIV Positive	4 (3.0)	1 (0.7)	0.21
TKA			
Comorbidity	Alcohol cohort, n (%)	Povidone cohort, n (%)	*p*-value
Rheumatoid Arthritis	18 (11.0)	11 (6.5)	0.18
Class III Obesity	12 (7.4)	23 (13.6)	0.08
Tobacco Use	19 (11.7)	16 (9.5)	0.59
Diabetes Mellitus	36 (22.1)	29 (17.2)	0.27
HIV Positive	0	1 (0.6)	1

**Table 2:** No differences were found in rate of complications or infection between alcohol wash and povidone wash groups in both TKA and THA patients.

THA				
	Alcohol Cohort, n (%)	Povidone Cohort, n (%)	Odds Ratio	95% Confidence Interval
Rate of Complication	6 (4.5)	5 (3.6)	0.796	0.237–2.673
Rate of Infection	1 (0.7)	2 (1.4)	1.942	0.174–21.667
TKA				
	Alcohol cohort, n (%)	Povidone cohort, n (%)	Odds Ratio	95% Confidence Interval
Rate of Complication	3 (1.9)	2 (1.2)	0.635	0.105–3.849
Rate of Infection	0	1 (0.6)	0.994	0.983–1.006

## Data Availability

Deidentified data will be made available upon request.
